# Editorial: The Influence of Crystal Size and Morphology on Framework Materials

**DOI:** 10.3389/fchem.2021.829906

**Published:** 2022-01-07

**Authors:** Claire L. Hobday, Simon Krause, Sven M. J. Rogge, Jack D. Evans, Hana Bunzen

**Affiliations:** ^1^ Centre for Science at Extreme Conditions and EaStCHEM School of Chemistry, The University of Edinburgh, Edinburgh, United Kingdom; ^2^ Nanochemistry Department, Max Planck Institute for Solid State Research, Stuttgart, Germany; ^3^ Center for Molecular Modeling (CMM), Ghent University, Ghent, Belgium; ^4^ Centre for Advanced Nanomaterials and Department of Chemistry, University of Adelaide, Adelaide, SA, Australia; ^5^ Chair of Solid State and Materials Chemistry, Institute of Physics, University of Augsburg, Augsburg, Germany

**Keywords:** metal-organic frameworks (MOFs), crystal growth, crystal size and morphology control, covalent-organic framework (COFs), soft porous crystals, flexible metal-organic frameworks

**FIGURE 1:**
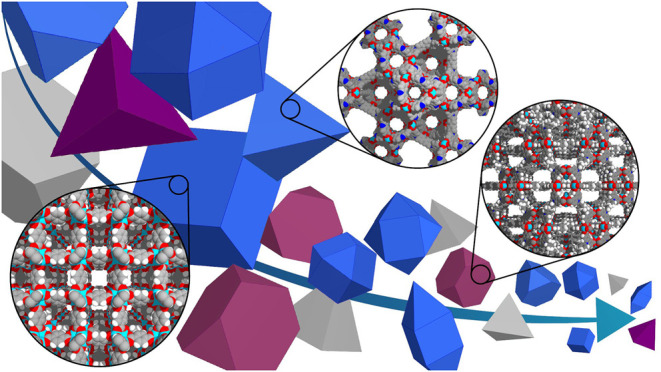
The size and morphology of framework materials may substantially impact macroscopic behaviours.

The current development of framework materials is generally achieved by modification of geometry and composition of the framework and building units. Why then, one may ask, is the emphasis of this Research Topic on characterizing framework materials with the same geometry and composition, which differ in seemingly unimportant characteristics such as particle size or morphology? Because, as demonstrated in recent works, parameters such as particle size distribution and morphology may have a major effect on material properties, as shown schematically in Figure 1. For example, different particle sizes of porous framework materials can influence the diffusion of guest molecules (i.e., pore accessibility), which has a direct impact on catalytic reactions, flexibility, and adsorption and separation processes.

In this collection of research articles, we highlight crystal size and morphology as a novel approach to modify the properties of materials. In particular, many dynamic metal-organic frameworks (MOFs) can be modified in this way, independent of composition and structure of the underlying material. As shown in this Research Topic, this recent insight has profound consequences in all aspects of functional material design and requires developing novel approaches and techniques, both from an experimental and a theoretical point of view, to fully understand and exploit this new parameter for material design.

In “Large-Scale Molecular Dynamics Simulations Reveal New Insights Into the Phase Transition Mechanisms in MIL-53(Al)”, Vandenhaute et al. address a first fundamental question in this regard by investigating crystal size effects on the breathing mechanism in the switchable MIL-53(Al) MOF. Thanks to their massive parallelization over state-of-the-art GPUs using the OpenMM software package and their implementation of a versatile pressure control algorithm, dynamic simulations of MIL-53(Al) cells of up to one million atoms came within reach. They adopted this approach to demonstrate that the MIL-53 transition mechanism depends on both the size and the mechanical pressure exerted on the material. At intermediate cell sizes and low pressures, the phase transition occurs *via* a layer-by-layer mechanism. In contrast, at higher pressures and sufficiently large unit cells, the phase transition is initiated at discrete nucleation points from which the transition propagates throughout the framework, temporarily leading to various phase domains in the system.

In “Molecular Dynamics Simulations of the Breathing Phase Transition of MOF Nanocrystallites II: Explicitly Modeling the Pressure Medium”, Schaper et al. demonstrate atomistic molecular dynamics simulations of transitions and energetic barriers for several pillared-layer MOF nanocrystallites, going beyond periodic boundary conditions. However, to treat the application of pressure, simulations of discrete systems cannot use the conventional description of pressure using periodic boundary conditions. Instead, the authors sought inspiration from the experimental practice of mercury nanoporosimetry. To apply pressure, a surrounding medium of particles was employed to transmit force. This new approach enabled atomistic simulations of the realistic pressure response of pillared-layer MOFs.

In “Impact of Crystal Size and Morphology on Switchability Characteristics in Pillared-Layer Metal-Organic Framework DUT-8(Ni)”, Abylgazina et al. demonstrate the change in adsorption-induced pore switching in a pillared-layer MOF as a function of crystal size and morphology. The authors identify that the gas pressure required for pore switching increases with decreasing crystal size and length. This contribution highlights the importance of experimental techniques to control particle size, *via* both reaction conditions and the introduction of modulators during the synthesis of the MOF, and to analyse their properties *via* correlation of scanning electron microscopy and adsorption behaviour.

Finally, in “Perspectives on the Influence of Crystal Size and Morphology on the Properties of Porous Framework Materials”, we briefly summarize the state of the art of crystal size phenomena with respect to mechanical and adsorption properties in addition to the importance of computational tools. We also indicate applications with respect to biomedical applications and provide a perspective of how the field could develop in the future.

